# Allogeneic hematopoietic stem cell transplantation in a patient with HIV-negative recurrent plasmablastic lymphoma: A case report

**DOI:** 10.1097/MD.0000000000024498

**Published:** 2021-02-19

**Authors:** Chunmeng Rong, Lixia Sheng, An Wu, Ye Sun, Guifang Ouyang

**Affiliations:** a Medical School of Ningbo University; b Ningbo First Hospital, Zhejiang, People's Republic of China.

**Keywords:** allogeneic hematopoietic stem cell transplantation, autologous stem cell transplantation, HIV-negative, plasmablastic lymphoma, relapsed

## Abstract

**Introduction:**

No standard guideline has been established for the treatment of plasmablastic lymphoma (PBL) and prognosis remains extremely poor, given that patients relapse early after chemotherapy and show resistance to commonly used cytostatic drugs.

**Patient concerns:**

We present the case of a 52-year-old HIV-negative man who presented with a mass at the left sternoclavicular joint. He had no significant comorbidities and no latent immunosuppression.

**Diagnosis:**

The largest lymph node measured was 36 × 19 mm. An excisional biopsy showed diffuse proliferation of large lymphoid cells which were positive for CD38 and CD138, but negative for CD20. He was diagnosed with stage IV PBL with a low IPI.

**Interventions:**

The patient was treated with four cycles of induction therapy with bortezomib, epirubicin and dexamethasone. He achieved complete remission. But 3 months after receiving consolidated autologous hematopoietic stem cell transplantation, he relapsed. Allogeneic hematopoietic stem cell transplantation was performed on the patient.

**Outcomes:**

The patient achieved remission again and there were no serious complications after allogeneic hematopoietic stem cell transplantation. This patient was followed up once every three months, and to date, he has been disease-free for more than 4 years.

**Conclusion:**

The survival of recurrent PBL after autologous hematopoietic stem cell transplantation is very poor. Salvage allogeneic hematopoietic stem cell transplantation may bring long-term survival opportunities for those patients. Further clinical studies are needed to explore the role of allogeneic hematopoietic stem cell transplantation in refractory and recurrent PBL.

## 1 Introduction

Plasmablastic lymphoma (PBL) is a rare and highly aggressive subtype of diffuse large B-cell lymphoma (DLBCL), characterized by plasma cell antigen differentiation. It was initially described in the setting of human immunodeficiency virus (HIV) infection, but it was also been reported in HIV-negative patients.^[1]^ There is no consensus on the standard of care for PBL. The role of intensification of induction chemotherapy is controversial. And novel agents, bortezomib and lenalidomide, have shown some effectiveness in relapsed cases and may have a relatively important role in frontline treatment.^[2,3]^ The use of autologous stem cell transplantation (ASCT) possibly improves outcomes when used as a consolidation or salvage therapy and may lead to better results than chemotherapy, but the available data remain sparse.^[2]^ The literatures on allogeneic hematopoietic stem cell transplantation (allo-HSCT) treatment for PBL are quite rare compared to ASCT. And given its rarity, most of the data available rely on case reports and case series. Patients with PBL have a poor prognosis, with median survival times shorter than 2 years.^[2,4]^ To the best of our knowledge, the survival of recurrent PBL is worse.^[2,5,6]^ Here, we report a case of HIV-negative PBL in a patient who had recurrence after ASCT, but achieved and maintained complete remission for 4-years after salvage allo-HSCT.

## 2 Case presentation

**Figure 1:**
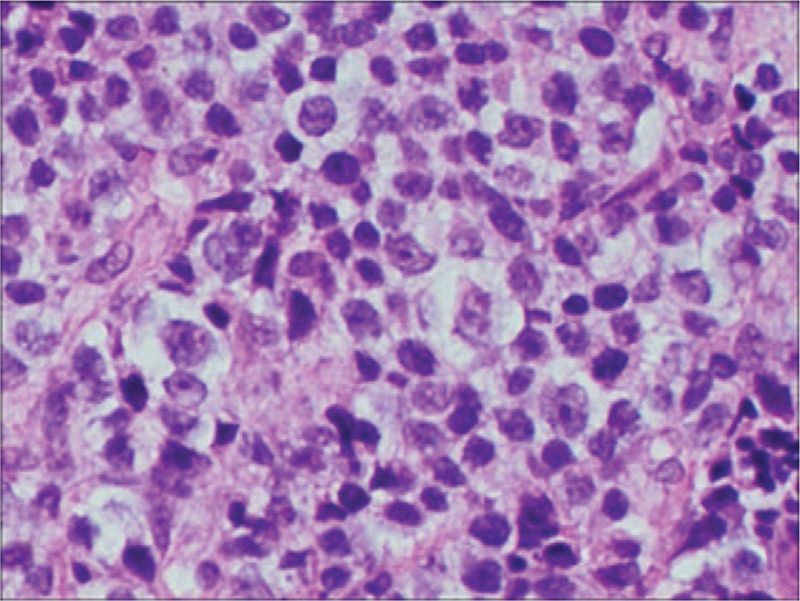
H&E (hematoxylin and eosin) image shows large sheets of mostly large plasmacytoid appearing mononuclear cells. (HE Magnification × 400).

**Figure 2:**
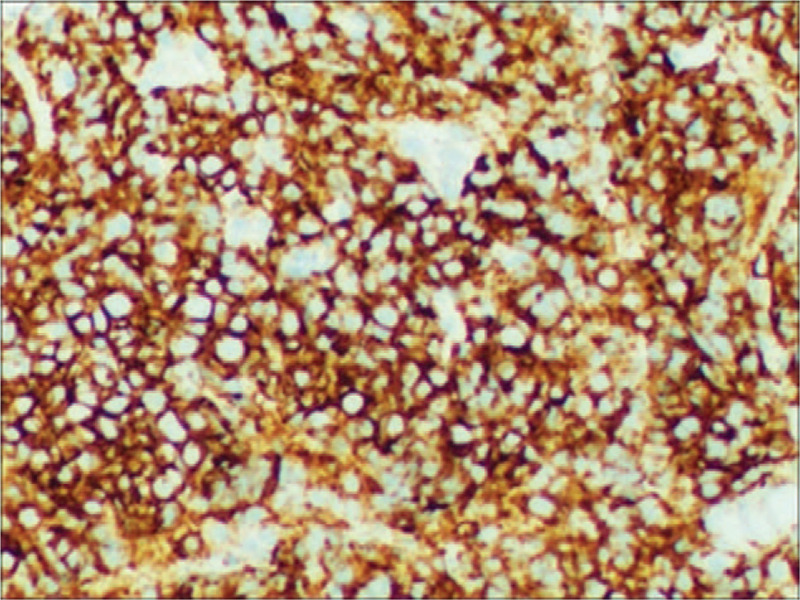
CD138, immunohistochemical stain demonstrates plasmacytic differentiation. (Magnification × 200).

**Figure 3:**
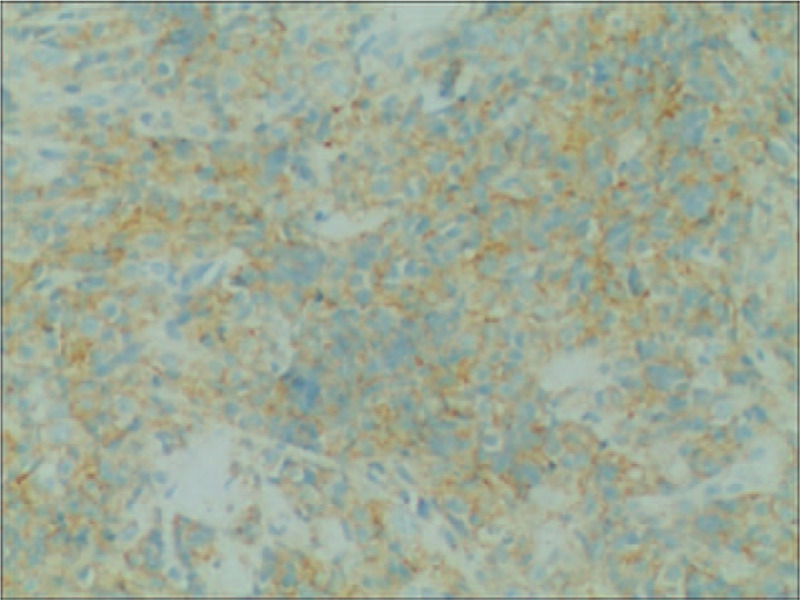
CD38, immunohistochemical stain demonstrates plasmacytic differentiation. (Magnification × 200).

**Figure 4:**
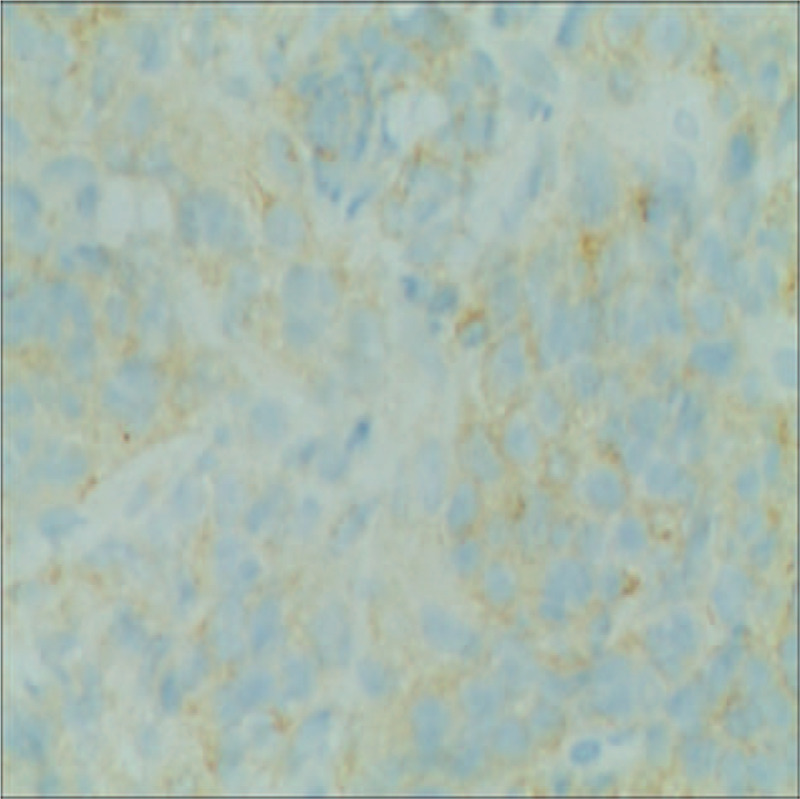
CD10, immunohistochemical stain demonstrates plasmacytic differentiation. (Magnification × 200).

**Figure 5:**
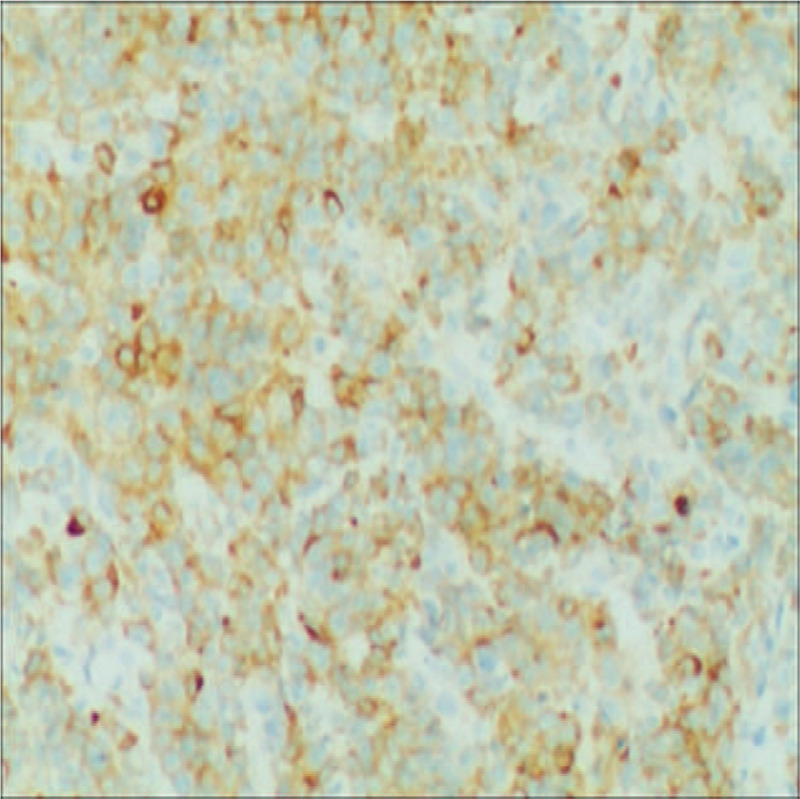
CD79a, immunohistochemical stain demonstrates plasmacytic differentiation. (Magnification × 200).

**Figure 6:**
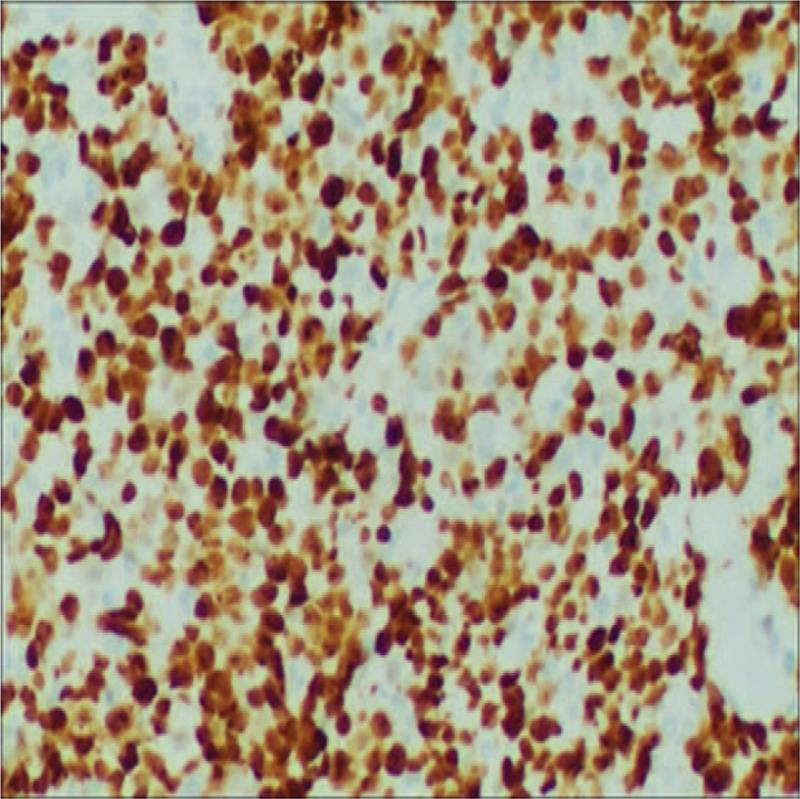
Strong and homogenous staining with Ki-67 indicating a high proliferation index. (Magnification × 200).

The patient was a 52-year-old man who presented in September 2015 with a mass at left sternoclavicular joint without any systemic symptoms. He had no significant comorbidities and no latent immunosuppression. Ultrasonography of lymph nodes indicated multiple lymph node enlargement in the neck, and multiple hypoechoic areas were seen above the left clavicle, the largest of which was 36 × 19 mm. A total body computed tomography (CT) scan showed that soft tissue mass shadows were seen around the left sternoclavicular joint and local cortical absorptions were present. Further investigations with fluorodeoxyglucose positron emission tomography–computed tomography (PET-CT) scan confirmed hypermetabolic mass appeared in several places of the body which was considered plasma cell infiltration. An excisional biopsy showed diffuse proliferation of large lymphoid cells (Fig. 1). Immuno-staining of the neoplastic cells showed negative for CD20, CD5, CD30, EREB, MPO, CD117 and Bcl-6, whereas it was positive for CD38, CD138, CD10, CD79a and Bcl-2 (Figs. 2–5). The Ki-67 proliferation index was 95% (Fig. 6). Biopsy of the iliac crest showed no marrow involvement. According to the above analysis, International Prognostic Index (IPI) scores were 2, the patient was diagnosed with stage IV PBL with a low IPI.

In addition, laboratory data were as following: Blood routine examination indicated that the white blood cells were 8.1 × 10^9^/L, the hemoglobin was 146 g/L and the platelets were 267 × 10^9^/L. Erythrocyte sedimentation rate, C-reactive protein, lactate dehydrogenase, β2 microglobulin, serum proteins and immunoglobulin levels were all within normal ranges. Immunofixation electrophoresis showed no monoclonal component. Human herpes virus 8 (HHV-8) and Epstein-Barr virus DNA (EBV-DNA) were negative. Besides, serology for HIV was also negative.

From September to December 2015, the patient received 4 cycles of PAD chemotherapy regimens: bortezomib was given at the dose of 2.4 mg/day on day 1, 4, 8, 11, epirubicin at the dose of 20 mg/day on days 1–3, and dexamethasone at the dose of 40 mg/day on days 1–4 and 8–11 of each 28-day cycle. Repeat PET/CT following 4 cycles of chemotherapy showed no abnormal flurodeoxyglucose metabolic foci and enlarged lymph nodes, thus being compatible with a complete response (CR).

Later on, peripheral blood stem cell (PBSC) mobilization was achieved by high-dose cyclophosphamide (2 g, day 1–3), etoposide (300 mg, day 1–3) and granulocyte colony-stimulating factor (G-CSF), and leukapheresis products containing a total of mononuclear cell (MNC) 9.78 × 10^8^/kg and CD34+ cells 6.9 × 10^6^/kg were collected. In April 2016, the patient received autologous hematopoietic stem cell transplantation as consolidation therapy. The conditioning regiments were melphalan (200 mg/m^2^ -2d) and bortezomib (1.75 mg -6, -3, +1, +4), and a total of 9.78 × 10^8^/kg MNC and 6.9 × 10^6^/kg CD34^+^ cells PBSC were reinfused. Platelets and white blood cells were recovered at 10 and 11 days after transplantation, respectively. The CT scan performed 1 month after ASCT confirmed a CR.

However, 3 months after ASCT, CT showed a new lesion appeared in the second rib on the right side, which was considered as relapse. After full assessment, the patient underwent HLA half-matched allo-HSCT in September 2016. The conditioning regimen was modified BuCy+ATG (cytarabine 3.5 g -10, -9; busulfan 51.6 mg Q6 h -8, -7, -6; cyclophosphamide 3 g -5, -4; MECCNU 450 mg -3; antithymocyte globulin 2.5 mg/Kg -5, -4, -3, -2), and the scheme for prevention of graft-versus-host disease (GVHD) was cyclosporin, mycophenolate mofetil and short-term methotrexate. The patient developed pulmonary fungal infection 20 days after allo-HSCT and took a turn for the better after treatment with voriconazole injection and voriconazole tablets. Unfortunately, the patient developed severe hemorrhagic cystitis 20 days later, but fortunately the condition improved after symptomatic treatment. There has been no evidence of acute or chronic GVHD. The patient achieved sustained complete donor engraftment as short tandem repeat monitoring was performed every three months after transplantation. A CR was metabolically and clinically confirmed by PET-CT scan after allo-HSCT. The patient is now healthy and in CR for 4 years after allo-HSCT.

## 3 Discussed

PBL is a distinct variant of diffuse large B-cell lymphoma initially described in HIV-positive patients.^[7]^ Nevertheless, PBL has also been described in HIV-negative individuals, particularly in association with potential immunosuppression such as after solid organ or bone marrow transplantation or with autoimmune disorders and malignanciess.^[8–11]^ HIV-negative PBL, in comparison with HIV-positive PBL, occurs in older patients, with less frequent involvement of oral mucosa or bone marrow and less frequently stages III-IV.^[3,12,13]^ A review of 114 HIV-negative patients with PBL, by Liu et al^[12]^ demonstrated that immunosuppression, MYC gene rearrangement, high-risk international prognostic index, and EBV negativity were poor prognostic factors. And the overall survival (OS) is between 9 and 19 months in HIV-negative patients.^[12]^ Similarly, a systematic review on 76 HIV-negative PBL patients showed median OS of 9 months with 2-year OS rate of 10%.^[13]^

Given its rarity and peculiar features, there is no standard of care for PBL and the treatment remains a challenge.^[11]^ The use of cyclophosphamide, doxorubicin, vincristine and prednisone (CHOP) and CHOP-like regimens are considered inadequate therapy, resulting short remission period,^[3,11]^ so the NCCN guidelines recommend more intensive regimens,^[14]^ including etoposide, vincristine and doxorubicin with bolus of cyclophosphamide and prednisone (EPOCH),^[15]^ cyclophosphamide, vincristine, doxorubicin, methotrexate alternating with ifosfamide, etoposide, cytarabine,^[16]^ or hyperfractionated cyclophosphamide, vincristine, doxorubicin and dexamethasone alternating with methotrexate and cytarabine.^[17]^ The benefit of ASCT in patients with PBL is not quite clear based on the limited data available. In a single institution report of 9 consecutive HIV-negative PBL patients, 7 patients achieved CR and 1 patient achieved partial response (PR) after chemotherapy.^[8]^ Four patients underwent consolidation with ASCT in first complete remission, and 2 of these patients were alive at median follow-up of 23.9 months. The third patient had disease recurrence at 14 months and the fourth patient had disease recurrence at 2 months after ASCT.^[8]^ Moffitt Cancer Center presented the experience of two HIV-negative patients with PBL in PR that underwent an ASCT, and two patients survived 6 and 12 months.^[12]^

Given the low efficacy of the standard therapy, and the poor outcome of these patients, there is a need to move toward new therapeutic strategies by incorporating new agents. Bortezomib is the most reported new drug for PBL and has been used as a single dose or in combination. Koji et al^[18]^ show the bortezomib and lenalidomide-based treatment was tolerated and the patient was continued with a PR for over 2 years. Bortezomib and lenalidomide have a certain therapeutic effect, and are mostly used in patients with refractory recurrence.^[18,19]^ There are few case reports showing single-agent lenalidomide or combination with other chemotherapeutic drug in refractory PBL.^[20–22]^ A dramatic response to Brentuximab has been reported in a single case, but the patient died shortly due to previous disease disabilities.^[23]^ A plasmablastic microlymphoma arising in HHV8-associated multicentric Castleman disease in an HIV-negative patient that showed a clinical response to siltuximab, an anti-IL6 antibody.^[24]^ The use of rituximab, a chimeric anti-CD20 monoclonal antibody, is not currently a therapeutic standard because the lack of CD20 expression by PBL cells, but combined with chemotherapy it could improve remission rates.^[3,25]^ Clinical trials with novel immunotherapeutic agents and other drugs targeting some genes involved in the activation of Nuclear factor kappa B pathways, some of which are already ongoing, may show promising results.^[18]^

The literature on allo-HSCT in PBL is limited compared with ASCT. Only few cases of allo-HSCT for PBL were reported, much less for recurrence PBL. Hamadani^[26]^ reported one patient with PBL in CR2 who underwent a reduced-intensity allogeneic stem cell transplantation, and was alive 2 years after transplantation. Liu et al^[8]^ reported one patient who had disease recurrence at 14 months after ASCT was treated with allo-HSCT. Despite consolidation with allo-HSCT, disease recurred 5 months after, and the patient died. In general, the efficacy of allo-HSCT in the treatment of HIV-negative PBL patients is still not ideal and the survival of recurrent PBL after allo-HSCT is very poor.

In our case, the patient had recurrence after consolidated ASCT. Then, he underwent the salvage allo-HSCT from his daughter and achieved good curative effect. The patient was in long-term complete remission and he is alive now. It may be attributed to his younger age, low IPI score and early allo-HSCT treatment after relapse. As far as we know, this is the first case of such a long survival time after allo-HSCT. For young relapsed patients with PBL, allo-HSCT may bring long-term survival opportunities. Due to the rarity of the disease, it is not feasible to carry out large-scale clinical trials. The evaluation of allogeneic hematopoietic stem cell transplantation in PBL patients requires more accumulation and sharing of clinical experience.

## Author contributions

**Data curation:** An Wu, Ye Sun.

**Funding acquisition:** Guifang Ouyang, Lixia Sheng.

**Methodology:** Lixia Sheng.

**Writing – original draft:** Chunmeng Rong.

**Writing – review & editing:** Chunmeng Rong.
